# What kills us and what moves us: A comparative discourse analysis of heart disease and breast cancer

**DOI:** 10.1177/2055207619844865

**Published:** 2019-05-01

**Authors:** Claire E O’Hanlon

**Affiliations:** 1 Greater Los Angeles Health System, US Department of Veterans Affairs, Los Angeles, CA, USA; 2 RAND Corporation, Department of Economics, Sociology and Statistics, Santa Monica, CA, USA

**Keywords:** Social media, health communication, discourse analysis, lexical analysis, lexicogrammatical analysis, breast neoplasms, breast cancer, heart diseases, chronic disease, female

## Abstract

**Introduction:**

Heart disease kills nearly 300,000 US women annually, while approximately 40,000 US women die of breast cancer. Breast cancer online patient communities are well known for their high engagement and emotional support. This exploratory study compared social media discourse on breast cancer with discourse related to heart disease.

**Methods:**

Computer-assisted text analysis of two corpora composed of Twitter posts using #BreastCancer and #HeartDisease hashtags from December 2013 to December 2014. Lexical analysis (word and hashtag level) used AntConc software and lexicogrammatical analysis (style and stance) was conducted with DocuScope.

**Results:**

The #BreastCancer corpus consisted of 592,046 posts, 57% of which were not original to the user (retweets). #HeartDisease had 269,769 posts (13% retweets). Social media discourse about #BreastCancer and #HeartDisease drew attention to women, new developments, appeals for help and disease risks. The #BreastCancer corpus incorporates gendered language and associations with art and activism, while posts about #HeartDisease were discussed scientifically in concert with other diseases. The #BreastCancer corpus uniquely included community-specific initialism hashtags. Stance analysis of the #BreastCancer corpus revealed more socially oriented posts, marked by language of constructive reasoning, inclusive language and abstract thought, while #HeartDisease corpus posts were more scholarly, used contingent and oppositional reasoning, language from institutional and academic registers, citations and meta-discourse.

**Conclusion:**

The #HeartDisease social media community is less engaged, and content is less specific to both the disease and individual experience than #BreastCancer. Cultivating a women-focused heart disease online community might replicate some of the #BreastCancer community’s successes.

## Introduction

Social media has become an important aspect of Americans’ everyday lives, and is increasingly a space where health and health care are discussed.^1,2^ Social media has the potential to help both understand,^3^ address^4^ and engage the public^4^ on long-standing public health challenges, such as chronic diseases. Online social networks have formed around disease communities; online communities focused on cancer and cardiovascular disease have historically had high membership and engagement.^5^ As the number one cause of death in the US is heart disease, a chronic condition that kills about 600,000 Americans every year,^6^ the need to develop better strategies to make progress on combating this disease is pressing.

Despite perceptions that heart disease is a men’s disease, heart disease kills only slightly more men than women each year (53% men to 47% women), and ‘major cardiovascular diseases’, which is more broadly defined than heart disease, kills even more women than men.^6^ However, heart disease kills 15 times as many Americans as the 40,000 deaths due to breast cancer annually, and heart disease is seven times more likely to kill a woman than breast cancer is. In spite of these odds, women are less worried about developing heart disease than developing breast cancer.^7^

Among patient communities, breast cancer patient, survivor and advocacy communities are especially notable for the high levels of emotional support they provide,^8^ far exceeding levels provided in online communities for men’s diseases like prostate cancer^9,10^ or in many disease community groups where the population affected is closer to gender parity.^8^ Breast cancer online support communities can improve knowledge, increase engagement with activism and outreach activities, and reduce anxiety among participants.^11^ These communities provide more than just emotional and informational support; online disease communities are also reflections of real interest groups that can have significant influence on health over the long run, such as through their influence on allocations of government funding for medical research^12^ and the perceived prevalence of a condition by the public.^13^ In addition, these communities can affect research and awareness through fundraising. The most successful single disease-focused fundraiser in the US, the Susan G. Komen Race for the Cure, raises approximately $250 million for breast cancer causes every year, over four times as much as the biggest single heart disease fundraiser, the American Heart Association’s Jump Rope for Heart, which raises $54 million a year.^14^

Given the high levels of attention and funding allocated toward breast cancer-related causes compared to those for heart disease, it is likely that other disease communities can learn from the breast cancer community about how to educate, activate and support heart disease patients, which can have an important impact on perceptions and policy decisions related to heart disease in the long run. This study aims to compare and contrast how the breast cancer and heart disease communities interact on social media through discourse analysis, to determine if and how social media can be better leveraged to educate, activate and support heart disease patients. In addition to comparing overall engagement, we use established discourse analysis techniques to determine common words, hashtags and rhetorical strategies used in social media posts to characterize the differences in how the breast cancer and heart disease communities communicate online.

## Methods

### Data sources

Two text data sets (‘corpora’) were generated from Twitter posts (‘tweets’) collected by Symplur’s Healthcare Hashtag Project.^15^ These corpora consist of tweets that incorporate one of two specific hashtags, ‘#BreastCancer’ and ‘#HeartDisease’. I chose to analyse tweets with these relatively generic hashtags because their use is not necessarily associated with being part of a social media disease community, fundraiser or other organized movement. Each corpus consisted of 1 years’ worth of tweets collected from December 2013 to December 2014. I chose to analyse 1 years’ worth of tweets to ensure my data set was large enough to provide sufficient variety and volume to conduct statistical tests, and ensure that tweets about relevant annual events, such as fundraisers and conferences, were covered.

### Data cleaning

I used two different analytical tools that interpret text differently, which necessitated creating two versions of each corpus. For both versions, I used search and replace functions for regular expressions in the free text editor Notepad++^16^ to remove hyperlinks and usernames that would not be of interest to this analysis, and to protect the privacy of users. The two corpora were then used for lexical analysis. For the version of each corpus on which I conducted the lexicogrammatical analysis, I removed the hashtag symbols (‘#’) and disaggregated common multiword strings that were joined as a hashtag. This allowed the text analysis software to ‘read’ hashtags (e.g. read #keepfighting as ‘keep fighting’).

### Lexical analysis

Lexical analyses was conducted in AntConc,^17^ a free concordance analysis software program that allows the user to observe frequencies of words, as well as more complex functions such as collocates and n-grams.^18^ I extracted the 15 most commonly used words (not including words that are common in almost any English text, like ‘with’ and ‘is’) in each corpus. I also looked at the most common hashtagged terms in each corpus.

### Lexicogrammatical analysis

Lexicogrammatical analysis to directly compare the two corpora was conducted in DocuScope,^19^ a computer-aided discourse analysis software program that allows the user to observe the relative frequencies of language features within a hierarchical taxonomy of representation (i.e. how speakers and writers represent relationships, values, certainty, intensity, emotion etc.).^20,21^ DocuScope analyses 118 language action types (LATs), which are subcategories of 43 dimensions, grouped into 15 clusters. To conduct statistical tests, each corpus was divided into chunks of 2000 words. Thus, each chunk contains Twitter posts from the same time period and consistency across chunks can be investigated, rather than just means across the entire corpus as a whole.

I used analysis of variance (ANOVA) tests to look at the frequencies of the LATs and determine whether use across the two corpora was significantly different. For corpora of this size, it is possible that the corpora would differ significantly on a few of the 118 language categories investigated by DocuScope by chance. Since I planned to conduct a large number of statistical comparisons and wanted a high level of confidence that differences in the corpora were not due to chance, I used the Bonferroni adjustment to set my a priori level of significance to 0.05/118 = 0.00042. Thus, I considered the result of any ANOVA test with a *p*-value < 0.00042 to be non-significant, which is a relatively conservative approach.

## Results

### Lexical analysis

A comparison of the volume and language variance in the #BreastCancer and #HeartDisease corpora is shown in Table 1. The #BreastCancer corpus was more than twice the size of the #HeartDisease corpus. The #BreastCancer corpus also included a much larger set of individual words (more than four times the number in the #HeartDisease corpus) in spite of having a higher volume of retweets (57% for #BreastCancer versus 13% for #HeartDisease).

**Table 1.** Comparison of volume and variance of #BreastCancer and #HeartDisease corpora.

	#BreastCancer corpus (*n*)	#HeartDisease corpus (*n*)
Posts (tweets)	592,046	269,769
Total words	7,750,129	2,789,827
Individual, non-repeated words	95,311	22,402
Instances of ‘RT’	338,940	34,519

RT: Indicates a ‘retweet’. This implies that the text was originally composed by someone else, and was copied and shared by another user.

The 15 most common words (excluding words common to any English text) in each corpus are shown in Table 2. Both corpora include the words ‘Women’, ‘Help’, ‘New’ and ‘Risk’ in their top 15 words. The high frequency of ‘Women’ in the #HeartDisease corpus indicates that there appears to be an effort on social media to draw attention to heart disease in women. Furthermore, both corpora appear to include calls for assistance (‘Help’) for those with the disease as well as educating people about the possibility that they will acquire the disease (‘Risk’).

**Table 2.** Fifteen most common words in #BreastCancer and #HeartDisease corpora.

#BreastCancer^a^ corpus	#HeartDisease^b^ corpus
*Help*	Prevent
Support	Prevention
Fight	Preventing
Awareness	*Risk*
Pink	*Help*
Order	*Women*
Art	Health
Project	Cancer
Bare	Study
Reality	*New*
*Women*	Stroke
*Risk*	Diabetes
Please	Healthy
*New*	Diet
Via	Death

Terms common to both corpora are italicized. RT: Indicates a ‘retweet’. This implies that the text was originally composed by someone else, and was copied and shared by another user.

^a^Excludes #BreastCancer, breast, cancer, hashtagged terms, RT and common English words (e.g. with, is and for).

^b^Excludes #HeartDisease, heart, disease, hashtagged terms, RT and common English words (e.g. with, is, and, for).

In addition, this table makes apparent some of the major differences in the two corpora. The #BreastCancerAwareness corpus tends to consist of calls to action and support for people with the disease (‘Support’, ‘Fight’ and ‘Awareness’), while the #HeartDisease corpus appears to be more informational regarding prevention and research (‘Prevent’/‘Prevention’/‘Preventing’, ‘Healthy’ and ‘Diet’). The #HeartDisease corpus appears to include information about other health problems (‘Stroke’, ‘Cancer’ and ‘Diabetes’). ‘Pink’ also shows up in the #BreastCancer corpus, but the heart disease colour (‘Red’) does not appear in the top 15 in the #HeartDisease corpus. ‘Via’ appears in the top of the #BreastCancer corpus, indicating increased sharing of news articles through Twitter buttons on webpages, which automatically inserts ‘via’ and the source into posts created using those features.

The differences in the most common words used in each corpus could also be considered female gendered, such as the obvious ‘Pink’, but also ‘Art’ or ‘Please’. The #BreastCancer corpus appears to include seemingly random words (e.g. ‘Bare’ and ‘Reality’), but they are associated with a project entitled *Bare Reality*, a book that came out in 2015 that collected stories about and photographs of breasts, and whose proceeds benefit a breast cancer charity^22^; a single Twitter post (‘Support this art project & help fight #BreastCancer Pre-order ‘Bare Reality' [link] #feminism #photogr’ related to this project was reposted nearly 40,000 times, constituting 6.6% of all #BreastCancer traffic and containing 8 of the 15 most common words in this corpus. In contrast, the #HeartDisease corpus has more scientific and neutrally gendered language (‘Study’, ‘Health’ and ‘Death’). The most reposted #HeartDisease post, ‘One heartbeat tells [name and handle]’s story of near-death & recovery from #HeartDisease [link]’ was only retweeted 6000 times, constituting only 2% of all #HeartDisease traffic and containing only 1 of the top 15 most common words. Highly retweeted #BreastCancer posts often had 2000–4000 retweets, whereas highly retweeted #HeartDisease posts had 100–200.

Table 3 shows the top 10 most commonly used hashtagged terms in each corpus. Both corpora include the hashtags #health and #cancer. The most common terms in each corpora include references to the symbolic colour association of the disease, with #pink for breast cancer and #GoRed (‘Go Red’) for heart disease. One stark difference is the presence of three initialisms in the #BreastCancer corpus: #BCSM (‘breast cancer social media’), #BCAM (‘breast cancer awareness month’) and #BCA (‘breast cancer awareness’). #BCSM is a hashtag used by a specific organization that has frequent ‘tweet chats’ on social media; its use indicates a certain degree of intimacy with the breast cancer patient and/or advocacy community. The other hashtags #BCAM and #BCA are more generically used, but their use still indicates increased awareness or an advocacy role among its users compared with a generic disease hashtag (like #BreastCancer or #cancer). As before, the #HeartDisease corpus is dominated by other generic disease hashtags, such as #diabetes and #stroke, as well as heart-specific terms like #HeartAttack, #HeartHealth, and associated words such as #cholesterol and #obesity.

**Table 3.** Ten most common hashtags in #BreastCancer and #HeartDisease corpora.

#BreastCancer corpus	#HeartDisease corpus
#BreastCancerAwareness	#diabetes
#HeartAttack	#stroke
#photogr	*#health*
*#cancer*	#heart
#pink	*#cancer*
#BCSM	#cholesterol
#awareness	#GoRed
*#health*	#obesity
#BCAM	#HeartHealth
#BCA	#HeartAttack

Hashtagged terms common to both corpora are italicized.

Again, the hashtagged terms also show some slightly gendered trends. The number two most common hashtag in the #BreastCancer corpus is #feminism. It also includes an art reference through #photogr, which is a photography hashtag; again, this is often associated with the *Bare Reality* project. The #HeartDisease corpus common hashtags are again more neutrally gendered.

### Lexicogrammatical analysis

I conducted ANOVA tests on all 118 LATs in DocuScope to determine if the use of each type was different in the two corpora. Despite choosing a conservative significance level, 78 of the 118 LATs were deemed to be significantly different across the two data sets using the Bonferroni-adjusted significance threshold, and only 40 were deemed to be non-significantly different between the two data sets. If the 0.05 significance level had been applied, only 21 LATs would have been non-significantly different and 97 would have been considered significantly different. I do not present results that I considered spurious or of low relevance. For example, the word ‘breast’, which is viewed as a ‘Personal Property’ (in the ‘Character’ cluster) by the DocuScope dictionary, obviously appears a lot in the #BreastCancer corpus compared to the #HeartDisease corpus. However, since other words indicating personal property rarely appear compared to ‘breast’, I considered this association to be an artefact of the specificity of the corpus. Instead, I present selected results on contrasting usage of different types of reasoning, the presence of inclusive language, talk about the past and future, and the use of institutional and academic registers. The #BreastCancer corpus’s use of constructive reasoning, inclusive language, talk about the past and future, and abstract thought indicate a community conversation that is personal and social, while the #HeartDisease corpus’s use of contingent and oppositional reasoning, as well as institutional and academic registers (especially citations and meta-discourse), indicate an impersonal, information-focused community conversation.

In the reasoning cluster, LATs in the constructive reasoning dimension were used more heavily in the #BreastCancer corpus, while contingent reasoning and oppositional reasoning were used more in the #HeartDisease corpus. Constructive reasoning includes words and phrases like ‘so’, ‘because’, ‘in support of’ and ‘my reason for’. Words like ‘can’, could’ and may are indicative of contingent reasoning, while oppositional reasoning is indicated by phrases like ‘do not’, ‘are not’, and ‘debunks’. Constructive reasoning in the #BreastCancer corpus appears to be used to indicate reasons for supporting breast cancer-related causes (‘I have breast cancer **so** my sisters and I are shaving our hair’), and relating personal stories of family members and friends with the disease (‘She cries, not **because** she is weak but **because** she has been strong for so long’). Contingent and oppositional reasoning are used in the #HeartDisease corpus to disseminate information about heart disease prevention and treatment (‘The flu vaccine **could be** the key to preventing heart disease’, ‘Find out why multivitamins **do not** prevent heart disease’). The differences in the use of reasoning point to the more informational nature of tweets about heart disease, compared to a more personal and persuasive use of reasoning in Twitter posts about breast cancer.

In the personal relationships language cluster, the overwhelming presence of inclusive language in the #BreastCancer corpus compared to the #HeartDisease corpus is pronounced. Examples of inclusive language are ‘community’, ‘our’, ‘us’, ‘together’ and ‘participating’. In addition, talking both about the past and future are much more pronounced in the #BreastCancer corpus. Discourse about the future includes talking about fundraising goals, ways of reducing or increasing the risk of developing breast cancer, and what future research in the field will be like. Some of the common talk about the past includes remembering people who have died of breast cancer as well as the milestones of survivors.

The #HeartDisease data set includes much more language from the institutional register and the academic register, especially the use of citations. The institutional register includes values that most people believe to be good (help, accuracy, peace) or bad (disorder, deficiency, ills), as well as words that refer to public or institutional commonplace authorities that are known and respected. The academic register includes many different LATs, ranging from presence of abstract thought (‘feminism’, ‘art’) to citations (‘tell’, ‘say’) and meta-discourse (use of cues to guide the reader or listener to what is important). The #HeartDisease corpus dominates both the use of citations and meta-discourse, which again points to the impersonal, informational nature of tweets in this corpus. The one dimension of the academic register where the #BreastCancer corpus dominates is abstract thought, which again reflects a less informational and more intimate view of the thoughts of breast cancer patients, survivors, family members and advocates.

## Discussion

Overall, this analysis makes apparent the differences in both the volume, engagement and content of social media discourse on breast cancer and heart disease. Breast cancer has a much larger patient and advocacy community, and this community amplifies messages more effectively. Taken together, language about reasoning, the past, future, inclusion, values, authority, citation and abstract thought about breast cancer compared to heart disease reflects the relatively personal and emotional social media presence of people within breast cancer social media communities, compared to the neutral outsider perspective in social media posts related to heart disease.

In contrast to breast cancer, people with heart disease represent a less bounded disease community. Not only is heart disease a catch-all term for a number of cardiac conditions, heart disease is just one of many chronic, lifestyle-related diseases that are lumped together in social media discourse. A potential avenue to better engage and activate heart disease patients, and advocates, might be to identify and target individual affinity groups within this disease community, such as women, rather than trying to lump all people and lifestyle-related diseases together. This may also help amplify the fact that gender plays a critical role in how several aspects of heart disease manifest,^23^ including risk factors,^24^ symptoms^25^ and treatment.^26^ By creating a space for social media discourse for women and heart disease alone, heart disease advocates may be able to replicate the strong emotional support and other positive effects of social media discourse in the breast cancer patient and advocacy communities.

This study is subject to several limitations. Social media posts are challenging to analyse with text analysis software,^27^ due to the presence of slang, abbreviations, purposeful and non-purposeful misspellings, non-English words, characters and emoji. It was infeasible to disaggregate all multiword strings used in hashtagged terms manually in a data set of this size, so both corpora still contained artefacts that would not be useful in a corpus analysis or be readable by software. Furthermore, only 1 years’ worth of Twitter posts were analysed, and that year may not be representative of social media activity in other years.

Although the ability of social media to impact human health remains somewhat elusive,^28^ the potential of social media for activating, educating, and engaging patient and disease communities is great. Future work should aim to more closely examine the relationship between social media use and health outcomes. In the meantime, public health practitioners may wish to learn how to best cultivate a vibrant and engaged online community like those related to breast cancer. These online social communities are likely a critical component for influencing the social factors^2^ that may address health behaviour-based chronic conditions like heart disease.

## Supplemental Material

Supplemental material for What kills us and what moves us: A comparative discourse analysis of heart disease and breast cancer Click here for additional data file. Supplemental Material for What kills us and what moves us: A comparative discourse analysis of heart disease and breast cancer by Claire E O’Hanlon in Digital Health

## Authors’ Note

The contents do not represent the views of the U.S. Department of Veterans Affairs or the United States Government.
