# Supplementary material for: What kills us and what moves us: A comparative discourse analysis of heart disease and breast cancer
Source: Digit Health. 2019 May 1;5:2055207619844865. doi: 10.1177/2055207619844865 (PMC6495432; doi:10.1177/2055207619844865)
Supplement: Supplemental material for What kills us and what moves us: A comparative discourse analysis of heart disease and breast cancer [file Supplemental_Material.pdf]

Appendix

The structure of DocuScope language clusters, dimensions, and language action types (LATs) are shown in the following list. Clusters are bold, dimensions are italicized, and LATs are normal text.

Subjective Register
- *First Person*
  - First Person
- *First Person Personal*
  - Self-Disclosure
  - Self-Reluctance
  - Autobiography
- *Personal Register*
  - Private Thinking
  - Disclosure
  - Intensity
  - Immediacy
  - Subjective Time
  - Subjective Perception
- *Confidence*
  - Confidence
- *Uncertainty*
  - Uncertainty

Emotion
- *Positive Emotion*
  - Positivity
- *Negative Emotion*
  - Negativity (spurious)
  - Anger
  - Fear
  - Sad
  - Reluctance
  - Apology

Description
- *Sensory Language*
  - Sense Property
  - Sense Object
- *Space Movement*
  - Space Relation
  - Scene Shift
  - Motions
- *Dialog Orality*
  - Dialog Cues
  - Oral Cues

Institutional Register

- *Public Sources*
    - In Media
    - Common Authorities
- *Public Responsibility*
    - Responsibility
- *Positive Values*
    - Standards Pos
    - Innovations
- *Negative Values*
    - Standards Neg (spurious - disease)

Academic Register

- *Abstract Thought*
    - Abstract Concepts (spurious)
    - Communicator Role
    - Lang Ref (spurious)
- *Citing Others*
    - Precedent Defending
    - Received POV
    - Confirmed Thought
    - Citations
    - Neg Citation
    - Repair Citation
    - Speculative Citation
    - Authoritative Citation
    - Contested Citation
    - Attack Citation
    - Quotation
- *Guide thru Complexity*
    - Meta-discourse

Future

- *Future*
    - Project Ahead
    - Predicted Future

Past

- *Past*
    - Project Back
    - Future in Past

Personal Relations

- *Positive Relations*
    - Positive Attribution
    - Promise
    - Self Promise
    - Reassure
    - Reinforce
    - Acknowledge
    - Accept Agree

Inclusive Relations
- Inclusive

Negative Relations
- Negative Attribution
- Negative Relation

Reasoning

Constructive Reasoning
- Reason Forward
- Reason Backward
- Direct Reasoning
- Support

Contingent Reasoning
- Contingency

Oppositional Reasoning
- Deny Disclaim
- Concessive
- Resistance

Interactive

Inquiry
- Curiosity
- Question
- Future Question
- Open Query

Addressing Other
- Attention Grabber
- You Attention
- You Reference
- Request
- Follow Up
- Feedback
- Pos Feedback
- Neg Feedback
- Prior Knowledge

Elaboration

General Example Except
- Generalization
- Example
- Exceptions

Comparison
- Comparison
- Resemblances

Defining Specifying
- Specifiers
- Definition
- Numbers

Reporting

- *Reporting States*
    - Reporting States
- *Reporting Events*
    - Reporting Events
- *Reporting Process*
    - Recurring Events
    - GenericEvents
    - Sequence
    - Mature Process
    - Cause
    - Consequence
- *Reporting Change*
    - Transformation
    - Substitution
    - Updates
    - Precedent Setting

Directing

- *Directives*
    - Imperative
    - Procedures
    - Move Body
    - Confirm Experience
    - Error Recovery
- *Insistence*
    - Insist
- *Prohibitive*
    - Prohibitive

Narrative

- *Narrative Verbs*
    - Narrative Verbs
- *Time Expressions*
    - Time Shift
    - Time Duration
    - Biographical Time
    - Time Date
- *Narrative Background*
    - Aside

Character

- *Personal Pronoun*
    - Person Pronoun
- *Person Attribution*
    - Neutral Attribution
- *Person Class*
    - Person Property
